# Role of aminoglycoside-modifying enzymes and 16S rRNA methylase (ArmA) in resistance of *Acinetobacter baumannii* clinical isolates against aminoglycosides

**DOI:** 10.1590/0037-8682-0599-2020

**Published:** 2021-01-29

**Authors:** Maryam Asadi Jouybari, Mohammad Ahanjan, Bahman Mirzaei, Hamid Reza Goli

**Affiliations:** 1 Mazandaran University of Medical Sciences, Faculty of Medicine, Molecular and Cell Biology Research Centre, Sari, Iran.; 2 Mazandaran University of Medical Sciences, Faculty of Medicine, Department of Medical Microbiology and Virology, Sari, Iran.; 3 Zanjan University of Medical Sciences, School of Medicine, Department of Medical Microbiology and Virology, Zanjan, Iran.

**Keywords:** Acinetobacter baumannii, Aminoglycoside-modifying enzymes, ArmA, Aminoglycoside resistance

## Abstract

**INTRODUCTION:**

This study aimed to determine the role of genes encoding aminoglycoside-modifying enzymes (AMEs) and 16S rRNA methylase (ArmA) in *Acinetobacter baumannii* clinical isolates.

**METHODS:**

We collected 100 clinical isolates of *A. baumannii* and identified and confirmed them using microbiological tests and assessment of the *OXA-51* gene. Antibiotic susceptibility testing was carried out using disk agar diffusion and micro-broth dilution methods. The presence of AME genes and *ArmA* was detected by PCR and multiplex PCR.

**RESULTS:**

The most and least effective antibiotics in this study were netilmicin and ciprofloxacin with 68% and 100% resistance rates, respectively. According to the minimum inhibitory concentration test, 94% of the isolates were resistant to gentamicin, tobramycin, and streptomycin, while the highest susceptibility (20%) was observed against netilmicin. The proportion of strains harboring the aminoglycoside resistance genes was as follows: *APH*(3′)-*VIa* (*aph*A6) (77%), *ANT*(2”)-*Ia* (*aad*B) (73%), *ANT*(3”)-*Ia* (*aad*A1) (33%), *AAC*(6′)-*Ib* (*aac*A4) (33%), *ArmA* (22%), and *AAC*(3)-*IIa* (*aac*C2) (19%). Among the 22 gene profiles detected in this study, the most prevalent profiles included *APH*(3′)-*VIa* + *ANT*(2”)-*Ia* (39 isolates, 100% of which were kanamycin-resistant), and *AAC*(3)-*IIa* + *AAC*(6′)-*Ib* + *ANT*(3”)-*Ia* + *APH*(3′)-*VIa* + *ANT*(2”)-*Ia* (14 isolates, all of which were resistant to gentamicin, kanamycin, and streptomycin).

**CONCLUSIONS:**

High minimum inhibitory concentration of aminoglycosides in isolates with the simultaneous presence of AME- and ArmA-encoding genes indicated the importance of these genes in resistance to aminoglycosides. However, control of their spread could be effective in the treatment of infections caused by *A. baumannii.*

## INTRODUCTION

*Acinetobacter baumannii*, living in the soil, the water, and different hospital environments, is an important opportunistic pathogen that causes nosocomial infections such as pneumonia, urinary tract infections, intravenous catheter-associated infections, and ventilation-associated infections, particularly in intensive care units^1^ ^-^ ^4^. The ability of this microorganism to remain in the hospital environment and to spread among the patients, along with their resistance to several antibiotics, are the main driving forces behind large-scale recurrent events in different countries^5^.

The major antibiotics used for the treatment of infections caused by this organism are beta-lactams, aminoglycosides, fluoroquinolones, and carbapenems; however, *A. baumannii* has shown different rates of resistance against these antimicrobial agents^6^ ^-^ ^8^. These infections are difficult, costly, and sometimes impossible to treat owing to the high ability of *A. baumannii* to acquire antibiotic resistance genes and the development of multidrug-resistant (MDR) strains^9^ ^,^ ^10^. Aminoglycosides are one of the main drugs used for the treatment of Acinetobacter infections^11^; however, recently, the resistance of *A. baumannii* to these antibiotics has also increased. Two main mechanisms of resistance to aminoglycosides are the alteration of the ribosome structure caused by mutations in the ribosomal 16S rRNA and the enzymatic resistance mechanism^12^. The enzymatic alteration of the aminoglycoside molecule at -OH or -NH_2_ groups by aminoglycoside-modifying enzymes (AMEs) is the most important resistance mechanism^12^ ^-^ ^14^. AMEs are classified into three major groups: aminoglycoside phosphotransferase (APH), aminoglycoside acetyltransferase (AAC), aminoglycoside nucleotidyltransferase (ANT), and aminoglycoside adenylyltransferase (AAD)^5^ ^,^ ^13^. Aminoglycoside acetyltransferases cause acetylation of the -NH_2_ groups of aminoglycosides at the 1, 3, 2', and 6' positions using acetyl coenzyme A as a donor substrate^15^. Aminoglycoside phosphotransferases phosphorylate the hydroxyl groups present in the structure of aminoglycosides at the 4, 6, 9, 3', 2'', 3'', and 7'' positions (seven different groups) with the help of ATP; the largest enzymatic group in this family is the APH(3′)-I group^16^. The proportion of strains harboring the *aph*A6 gene in *A. baumannii* is widespread, and this enzyme is the cause of resistance to neomycin, amikacin, kanamycin, paromomycin, ribostamycin, butirosin, and isepamicin^17^. Aminoglycoside nucleotidyltransferases are classified into 5 groups, and the genes encoding these enzymes can be found in chromosomes or transferred by plasmids and transposons^12^. These enzymes transfer an AMP group from ATP to a hydroxyl group at the 2'', 3'', 4', 6, and 9 positions of the aminoglycoside molecule^13^. In addition to AMEs, 16S rRNA methylation by the ArmA enzyme is a novel mechanism that contributes to the high level of aminoglycoside resistance in *A. baumannii*, as reported in the Far East, Europe, and North America^5^. This enzyme can be transferred by class 1 integrons and is often detected in carbapenem-resistant *A. baumannii* isolates^18^. This study aimed to investigate the role of some important aminoglycoside-modifying enzymes and 16S rRNA methylase (ArmA) in the resistance of *A. baumannii* clinical isolates to aminoglycosides in Sari, located north of Iran.

## METHODS

### Sample collection and bacterial isolates

This study was performed on *A. baumannii* isolated from patients admitted to different educational hospitals in Sari, north of Iran, for 6 months (April 2019 to September 2019). The clinical specimens included blood, urine, respiratory secretions (bronchial lavage and tracheal secretions), CSF, and ulcer (surgical and burn wound). The clinical isolates were identified using conventional microbiological tests^19^ and confirmed by polymerase chain reaction (PCR) amplification of the *blaOXA*-51 gene using specific primers^20^; the reaction conditions are shown in Table 1. 

**TABLE 1:** Primers used to amplify the *blaOXA*-51 and aminoglycoside resistance genes along with the conditions of PCR.

Target genes	Primer sequences (5´-3´)	Amplicon size (bp)	94 °C	94 °C	Annealing Temperature and time	72 °C	72 °C	Reference
*OXA*-51	TAATGCTTTGATCGGCCTTG	353	2 min	25 sec	51 °C for 30 sec	30 sec	5 min	5
	TGGATTGCACTTCATCTTGG							
***APH*(3′)-*VIa***	CGGAAACAGCGTTTTAGA	717	2 min	25 sec	49 °C for 30 sec	30 sec	5 min	5
**(*aph*A6)**	TTCCTTTTGTCAGGTC							
***AAC*(3)-*IIa***	ATGCATACGCGGAAGGC	822	2 min	25 sec	54 °C for 30 sec	30 sec	5 min	5
**(*aac*C2)**	TGCTGGCACGATCGGAG							
***AAC*(6′)-*Ib***	TATGAGTGGCTAAATCGAT	395	2 min	25 sec	54 °C for 30 sec	30 sec	5 min	5
**(*aac*A4)**	CCCGCTTTCTCGTAGCA							
***ANT*(2")-*Ia***	ATCTGCCGCTCTGGAT	405	2 min	25 sec	49 °C for 30 sec	30 sec	5 min	5
**(*aad*B)**	CGAGCCTGTAGGACT							
***ANT*(3")-*Ia***	ATGAGGGAAGCGGTGATCG	792	2 min	25 sec	62 °C for 30 sec	30 sec	5 min	5
**(*aad*A1)**	TTATTTGCCGACTACCTTGGT							
*ArmA*	ATTCTGCCTATCCTAATTGG	315	2 min	25 sec	49 °C for 30 sec	30 sec	5 min	5
	ACCTATACTTTATCGTCGTC							

### Antimicrobial susceptibility testing

The antibiotic susceptibility pattern of the isolates was determined by the disk agar diffusion method on Muller Hinton agar (Merck, Germany) according to the Clinical and Laboratory Standards Institute (CLSI) guidelines^21^. The antibiotics included piperacillin (100 µg), piperacillin-tazobactam (100/10 µg), imipenem (10 µg), meropenem (10 µg), doripenem (10 µg), ciprofloxacin (5 µg), levofloxacin (5 µg), trimethoprim-sulfamethoxazole (1.25-23.75 µg), ceftazidime (30 µg), cefotaxime (30 µg), and cefepime (30 µg) (MAST Co., England). The susceptibility pattern of the isolates against aminoglycosides including kanamycin, amikacin, spectinomycin, netilmicin, gentamicin, streptomycin, and tobramycin was determined using the micro-broth dilution method according to the CLSI guidelines^21^. For interpretation of the minimum inhibitory concentration (MIC) values, we referred to the CLSI guidelines and previous studies^1^ ^,^ ^21^ ^,^ ^22^. *Escherichia coli* ATCC 25922 and *A. baumannii* ATCC 19606 were used as control strains for antibiotic susceptibility testing. 

### DNA extraction, PCR, and multiplex-PCR

DNA was extracted from all *A. baumannii*isolates grown for 24 h using an alkaline lysis method with sodium dodecyl sulphate (SDS) and NaOH, as previously published^23^, with few modifications. In brief, first, we prepared a lysis buffer by dissolving 0.5 g of SDS and 0.4 g of NaOH in 200 µL of distilled water. Next, 4-6 colonies of the bacteria were suspended in 60 µL of lysis buffer and subsequently heated at 95 °C for 10 min. In the next step, the suspension was centrifuged at 13000 rpm for 5 min, and 180 µL of distilled water was added to the microtubes. The obtained supernatant was frozen at −20 °C until use as the extracted DNA in PCR. 

Two sets of multiplex-PCR were used to detect AME-encoding genes in *A. baumannii* isolates using the specific primers shown in Table 1. *APH*(3′)-*VIa* (*aph*A6), *ANT*(2")-*Ia* (*aad*B), and *ArmA* genes were detected in the same set; *AAC*(6′)-*Ib* (*aac*A4) and *AAC*(3)-*IIa* (*aac*C2) were identified in the second set; and the *ANT*(3")-*Ia* (*aad*A1) gene was detected by PCR alone. The PCR and multiplex-PCR were performed in 25 µL of final volume containing 12.5 µL of the master mix (Ampliqon, Denmark), 10 pmol of each primer (Bioneer, South Korea), and 500 ng of template DNA; the reaction solutions were brought to the desired volume through the addition of distilled water. The genes were amplified under standard conditions using a thermocycler machine (Bio-Rad, USA). All reactions were performed in 34 cycles, and the conditions are shown in Table 1.

### Statistical analysis

The data were analyzed using SPSS (version 21). Categorical data were analyzed using the Fisher’s exact test, and a P-value less than 0.05 was considered statistically significant. In addition, an independent t-test was used to examine the mean age of the subjects. 

## RESULTS

### Patients, samples, and bacterial isolates

In this study, 100 non-duplicated *A. baumannii* clinical isolates were collected from 100 patients admitted to the teaching and educational hospitals of Sari, north of Iran. All isolates identified using the phenotypic method contained the *blaOXA*-51 gene according to the PCR results. The mean age of the patients was 42.08±25.08 years (minimum age: 6 months; highest age: 88 years), and 50% of the patients were male. There was no significant difference between men and women in terms of mean age (p=0.64). Most of the bacterial isolates (34%) were obtained from patients admitted to the burn wards, while 29%, 21%, and 16% of the isolates were collected from the ICU, surgery, and pediatric wards, respectively. The most common type of specimen (73%) for isolation of the bacteria was the wound samples; however, 15% and 12% of other clinical isolates were obtained from urine and blood cultures, respectively.

### Antimicrobial susceptibility pattern

According to the results of the disk agar diffusion method, the most and least effective antibiotics in the present study were imipenem and ciprofloxacin, with resistance rates of 75% and 100%, respectively (Table 2). Moreover, 94% of the isolates were detected as multi-drug resistant (MDR), and the most MDR isolates were collected from wound samples. Table 2 presents the antibiotic resistance patterns of all *A. baumannii* clinical isolates in this study based on hospital wards, as well as sample types. Resistance to the tested antibiotics was not significantly correlated with the sample types and hospital wards where the samples were collected.

**TABLE 2:** Antimicrobial susceptibility pattern of the *Acinetobacter baumannii* clinical isolates in disk agar diffusion method.

Antibiotics	No. (%) of resistant, intermediate resistant or susceptible isolates in terms of										
	Susceptibility	Total	Hospital wards					Sample types			
		(n=100)	Burn	ICU	Surgery	Pediatric	**P-*value***	Wound	Urine	Blood	**P-*value***
			(n=34)	(n=29)	(n=21)	s (n=16)		(n=73)	(n=15)	(n=12)	
PIP	R	86	28 (82.3)	28 (96.5)	19 (90.4)	11 (68.7)	0.412	62 (84.9)	14 (93.3)	10 (83.3)	0.917
	I	10	5 (14.7)	1 (3.4)	2 (9.5)	2 (12.5)		8 (10.9)	1 (6.6)	1 (8.3)	
	S	4	1 (2.9)	0	0	3 (18.7)		3 (4.1)	0	1 (8.3)	
PIP-TAZ	R	78	26 (76.4)	24 (82.7)	16 (76.1)	12 (75)	0.104	54 (73.9)	14 (93.3)	10 (83.3)	0.372
	I	10	4 (11.7)	2 (6.8)	3 (14.2)	1 (6.2)		8 (10.9)	0	2 (16.6)	
	S	12	4 (11.7)	3 (10.3)	2 (9.5)	3 (18.7)		11 (15)	1 (6.6)	0	
CAZ	R	76	25 (73.5)	22 (75.8)	18 (85.7)	11 (68.7)	0.743	52 (71.2)	15 (100)	9 (75)	0.559
	I	0	0	0	0	0		0	0	0	
	S	24	9 (26.4)	1 (3.4)	3 (14.2)	5 (31.2)		21 (28.7)	0	3 (25)	
CTX	R	93	32 (94.1)	28 (96.5)	19 (90.4)	14 (87.5)	0.762	67 (91.7)	15 (100)	11 (91.6)	0.618
	I	0	2 (5.8)	1 (3.4)	2 (9.5)	2 (12.5)		6 (8.2)	0	1 (8.3)	
	S	7	0	0	0	0		0	0	0	
CEF	R	92	32 (94.1)	28 (96.5)	18 (85.7)	14 (87.5)	0.448	67 (91.7)	14 (93.3)	11 (91.6)	0.728
	I	4	1 (2.9)	1 (3.4)	2 (9.5)	0		2 (2.7)	1 (6.6)	1 (8.3)	
	S	4	1 (2.9)	0	1 (4.7)	2 (12.5)		4 (5.4)	0	0	
IMI	R	75	27 (79.4)	25 (86.2)	17 (80.9)	10 (62.5)	0.617	55 (75.3)	12 (80)	8 (66.6)	0.873
	I	11	4 (11.7)	2 (6.8)	2 (9.5)	3 (18.7)		9 (12.3)	1 (6.6)	1 (8.3)	
	S	14	7 (20.5)	2 (6.8)	2 (9.5)	3 (18.7)		9 (12.3)	2 (13.3)	3 (25)	
MER	R	97	33 (97)	28 (96.5)	20 (95.2)	16 (100)	0.964	70 (95.8)	15 (100)	12 100)	0.667
	I	0	0	0	0	0		0	0	0	
	S	3	1 (2.9)	1 (3.4)	1 (4.7)	0		3 (4.1)	0	0	
DOR	R	96	32 (94.1)	28 (96.5)	20 (95.2)	16 (100)	0.797	69 (94.5)	15 (100)	12 (100)	0.913
	I	2	1 (2.9)	1 (3.4)	0	0		2 (2.7)	0	0	
	S	2	1 (2.9)	0	1 (5)	0		2 (2.7)	0	0	
CIP	R	100	34 (100)	29 (100)	21 (100)	16 (100)	0.100	73 (100)	15 (100)	12 (100)	0.100
	I	0	0	0	0	0		0	0	0	
	S	0	0	0	0	0		0	0	0	
LEV	R	93	31 (91.1)	27 (93.1)	20 (95.2)	15 (93.7)	0.725	67 (91.7)	14 (93.3)	12 (100)	0.842
	I	3	2 (5.8)	0	0	1 (6.2)		3 (4.1)	0	0	
	S	4	1 (2.9)	2 (6.8)	1 (4.7)	0		3 (4.1)	1 (6.6)	0	
SXT	R	92	31 (91.1)	27 (93.1)	18 (85.7)	16 (100)	0.935	68 (93.1)	12 (80)	12 (100)	0.216
	I	3	1 (2.9)	1 (3.4)	1 (4.7)	0		1 (1.3)	2 (13.3)	0	
	S	5	2 (5.8)	1 (3.4)	2 (9.5)	0		4 (5.4)	1 (6.6)	0	

**PIP:** piperacillin; **PIP-TAZ:** piperacillin-tazobactam; **CAZ:** ceftazidime; **CTX:** cefotaxime; **CEF:** cefepime; **IMI:** imipenem; **MER:** meropenem; **DOR:** doripenem; **CIP:** ciprofloxacin; **LEV:** levofloxacin; **SXT:** trimethoprim-sulfamethoxazole; **R:** resistant; **I:** intermediate resistant; and **S:** susceptible.

Moreover, according to the MIC results, the resistance rate against gentamicin, kanamycin, tobramycin, and streptomycin was 94%, while the highest susceptibility (20%) of *A. baumannii* isolates was observed against netilmicin. In contrast, 74%, 68%, and 78% of our clinical isolates were resistant to amikacin, netilmicin, and spectinomycin, respectively. The MIC ranges of aminoglycosides and their relationship with the presence of AMEs-encoding genes are shown in Table 3.

**TABLE 3:** Aminoglycoside resistance pattern of the *Acinetobacter baumannii* clinical isolates in this study.

Antibiotics	MIC ranges	No. of the isolates with							
	(µg/mL)	Susceptibility	***APH*(3′)-*VIa***	***ANT*(2")-*Ia***	***ANT*(3")-*Ia***	***AAC*(6′)-*Ib***	***AAC*(3)-*IIa***	*armA*	Total
		pattern	**(*aph*A6)**	**(*aad*B)**	**(*aad*A1)**	**(*aac*A4)**	**(*aac*C2)**	(n=22)	
			(n=77)	(n=73)	(n=33)	(n=33)	(n=19)		
Gentamicin	≤4	S	-	-	-	-	-	-	6
	8	I	5	3	1	1	-	1	-
	16-32	R	-	-	-		-	-	94
	64-128	R	5	-	-	-	-	-	
	≥256	R	67	70	32	32	19	21	
	MIC_50_		512	256	512	512	256	1024	256
	MIC_90_		1024	256	1024	256	256	1024	512
Tobramycin	≤4	S	3	3	-	-	-	1	4
	8	I	1	-	-	-	-	2	2
	16-32	R	2	-	1	-	-	-	94
	64-128	R	-	2	1	4	2	3	
	≥256	R	71	68	31	29	17	16	
	MIC_50_		256	512	512	256	512	512	512
	MIC_90_		1024	1024	1024	512	1024	512	1024
Amikacin	≤16	S	13	10	6	6	2	3	16
	32	I	7	10	6	6	5	1	10
	64	R	-	1	-	-	-	-	74
	128	R	-	4	-	-	-	-	
	≥256	R	57	48	21	21	12	18	
	MIC_50_		256	1024	512	512	512	256	512
	MIC_90_		512	512	512	1024	512	256	1024
Netilmicin	≤8	S	8	8	3	-	1	2	20
	16	I	6	-	-	4	-	6	12
	32	R	8	10	4	6	4	2	68
	64-128	R	5	7	1	3	2	4	
	≥256	R	50	48	25	20	12	8	
	MIC_50_		256	128	64	256	256	128	128
	MIC_90_		256	128	64	512	512	128	256
Kanamycin	≤16	S	4	2	-	-	-	-	4
	32	I	2	2	-	-	-	-	2
	64	R	-	3	-	1	-	-	94
	128	R	3	2	2	-	2	1	
	≥256	R	68	64	31	32	17	21	
	MIC_50_		64	32	128	64	256	128	128
	MIC_90_		64	256	256	64	256	256	256
Streptomycin	≤4	S	1	-	1	1	-	-	6
	8	S	1	1	-	1	-	-	
	16	S	-	-	-	-		-	
	32	I	-	-	-	-	-	-	-
	64-128	R	3	3	-	3	2	-	94
	≥256	R	72	69	32	28	17	18	
	MIC_50_		256	128	256	256	256	512	256
	MIC_90_		256	128	512	1024	128	512	512
Spectinomycin	≤4	S	1	1	-	-	-	3	12
	8	S	7	5	2	2	-	-	
	16	S		-		-	-	-	
	32	I	8	3	-	1	-	5	10
	64-128	R	2	1	-	2	2	1	78
	≥256	R	59	63	31	28	17	13	
	MIC_50_		256	256	512	64	64	256	256
	MIC_90_		256	512	256	128	128	256	512

**R:** resistant; **I:** intermediate resistant; **S:** susceptible.

Notes: **MIC**_50_ **:** Minimum inhibitory concentration required to inhibit the growth of 50% of organisms; **MIC**_90:_ Minimum inhibitory concentration required to inhibit the growth of 90% of organisms.

### Gene profiles of the isolates

The frequency of each aminoglycoside resistance gene and its relation with the MIC ranges are shown in Table 3. In total, the proportions of aminoglycoside resistance genes among our clinical isolates of *A. baumannii* were as follows: *APH*(3′)-*VIa* (*aph*A6) (77%), *ANT*(2")-*Ia* (*aad*B) (73%), *ANT*(3")-*Ia* (*aad*A1) (33%), *AAC*(6′)-*Ib* (*aac*A4) (33%), *AAC*(3)-*IIa* (*aac*C2) (19%), and *ArmA* (22%). The relationship between the presence of aminoglycoside resistance genes and the aminoglycoside susceptibility pattern of the isolates is shown in Table 4. There was a significant association between the presence of all resistance genes and the non-susceptibility (resistance or intermediate resistance) to all aminoglycosides, except *armA* and resistance to netilmicin. Important data from this table indicates that in some groups, such as gentamicin- and tobramycin-resistant groups, all resistant isolates contained some AMEs-encoding genes such as *aacC2*, *aacA4*, and *aadA1*. 

**TABLE 4:** The relationship between the presence of aminoglycoside resistance genes and the aminoglycoside susceptibility pattern of the *A. baumannii* clinical isolates.

Antibiotics	Susceptibility Pattern	No. (%) of the isolates contained											
		***APH*(3′)-*VIa* (*aph*A6)**	**P-*value***	***ANT*(2")-*Ia* (*aad*B)**	**P-*value***	***ANT*(3")-*Ia* (*aad*A1)**	**P-*value***	***AAC*(6′)-*Ib* (*aac*A4)**	**P-*value***	***AAC*(3)-*IIa* (*aac*C2)**	**P-*value***	*armA*	**P-*value***
		(n=77)		(n=73)		(n=33)		(n=33)		(n=19)		(n=22)	
Gentamicin	Non-susceptible	77 (100)	0.012	73 (100)	0.023	33 (100)	0.033	33 (100)	0.033	19 (100)	0.007	22 (100)	0.021
	Susceptible	0		0		0		0		0		0	
Tobramycin	Non-susceptible	74 (96.1)	0.019	70 (95.8)	0.029	33 (100)	0.003	33 (100)	0.003	19 (100)	0.007	21 (95.4)	0.024
	Susceptible	3 (3.8)		3 (4.1)		0		0		0		1 (4.5)	
Amikacin	Non-susceptible	64 (83.1)	0.036	63 (86.3)	0.037	27 (81.8)	0.038	27 (81.8)	0.038	17 (89.4)	0.024	19 (86.3)	0.033
	Susceptible	13 (16.8)		10 (13.6)		6 (18.1)		6 (18.1)		2 (10.5)		3 (13.6)	
Netilmicin	Non-susceptible	63 (81.8)	0.042	65 (89.04)	0.039	30 (90.9)	0.014	29 (87.8)	0.032	18 (94.7)	0.018	14 (63.6)	0.072
	Susceptible	15 (19.4)		8 (10.9)		3 (9.09)		4 (12.1)		1 (5.2)		8 (36.3)	
Kanamycin	Non-susceptible	73 (94.8)	0.029	71 (97.2)	0.023	33 (100)	0.003	33 (100)	0.003	19 (100)	0.007	22 (100)	0.021
	Susceptible	4 (5.1)		2 (2.7)		0		0		0		0	
Streptomycin	Non-susceptible	75 (97.4)	0.015	72 (98.6)	0.019	32 (96.9)	0.019	31 (93.9)	0.021	19 (100)	0.007	18 (81.8)	0.044
	Susceptible	2 (2.5)		1 (1.3)		1 (3.03)		2 (6.06)		0		4 (18.1)	
Spectinomycin	Non-susceptible	69 (89.6(	0.028	67 (91.7)	0.037	31 (93.9)	0.021	31 (93.9)	0.021	19 (100)	0.007	19 (86.3)	0.033
	Susceptible	8 (10.3)		6 (8.2)		2 (6.06)		2 (6.06)		0		3 (13.6)	

In addition, we detected 22 gene profiles among all clinical isolates of *A. baumannii* (Table 5). The most prevalent combination gene profiles in the present study included: 1) *APH(3')-VIa* + *ANT(2")-Ia* with 39 isolates containing these genes, among which 100% isolates were resistant towards kanamycin, while almost 95% were resistant against netilmicin and 97.4% were resistant to tobramycin and gentamicin, and 2) *AAC(3)-IIa* + *AAC(6')-Ib* + *ANT(3")-Ia* + *APH(3')-VIa* + *ANT(2")-Ia* with 14 isolates, among which 100% were resistant to gentamicin, kanamycin, and streptomycin, while almost 93% were resistant against tobramycin and spectinomycin. However, 15 isolates showed an AME gene profile with one AME gene, most of which were resistant to tested aminoglycosides. Other AME-encoding gene profiles were detected at a low rate (Table 5). However, 15, 52, 12, 5, 14, and 2 isolates in the present study contained 1, 2, 3, 4, 5, and 6 AME genes, respectively. The most prevalent gene profiles exhibited the simultaneous presence of 2 genes followed by 5 genes and 3 AME genes.

**TABLE 5:** Gene profiles of the *Acinetobacter baumannii* clinical isolates and their association with aminoglycoside resistance.

Gene profile (No.)	Gentamicin		Tobramycin		Amikacin		Netilmicin		Kanamycin		Streptomycin		Spectinomycin	
	NS	S	NS	S	NS	S	NS	S	NS	S	NS	S	NS	S
	(%)	(%)	(%)	(%)	(%)	(%)	(%)	(%)	(%)	(%)	(%)	(%)	(%)	(%)
*ArmA* (9)	9 (100)	-	9 (100)	-	7 (77.7)	2 (22.2)	3 (33.3)	6 (66.6)	9 (100)	*-*	7 (77.7)	2 (22.2)	9 (100)	-
*aphA6* (2)	2 (100)	-	2 (100)	-	-	2 (100)	-	2 (100)	1 (50)	1 (50)	2 (100)	-	-	2 (100)
*aadB* (4)	4 (100)	-	3 (75)	1 (25)	3 (75)	1 (25)	2 (50)	2 (50)	3 (75)	1 (25)	4 (100)	-	3 (75)	1 (25)
*ArmA + aphA6* (3)	3 (100)	-	3 (100)	-	2 (66.6)	1 (33.3)	2 (66.6)	1 (33.3)	3 (100)	*-*	3 (100)	-	2 (66.6)	1 (33.3)
*aacA4 + aadA1* (2)	2 (100)	-	2 (100)	-	2 (100)	-	1 (50)	1 (50)	2 (100)	*-*	2 (100)	-	2 (100)	-
*aadA1 + armA* (2)	2 (100)	-	2 (100)	-	1 (50)	1 (50)	2 (100)	-	2 (100)	*-*	1 (50)	1 (50)	2 (100)	-
*aadA1 + aphA6* (1)	1 (100)	-	1 (100)	-	1 (100)	-	1 (100)	-	1 (100)	-	1 (100)	-	1 (100)	-
*aadB + aadA1* (2)	2 (100)	-	2 (100)	-	2 (100)	-	2 (100)	-	2 (100)	*-*	2 (100)	-	2 (100)	-
*aphA6 + aadB* (39)	38 (97.4)	1 (2.56)	38 (97.4)	1 (2.56)	34 (87.1)	5 (12.8)	37 (94.8)	2 (5.1)	39 (100)	-	33 (84.6)	6 (15.3)	32 (82)	7 (17.9)
*aacA4 + aphA6* (3)	3 (100)	-	3 (100)	-	3 (100)	-	3 (100)	-	3 (100)	*-*	3 (100)	-	3 (100)	-
*aadB + aadA1 +* *aphA6* (2)	2 (100)	-	2 (100)	-	2 (100)	-	2 (100)	-	2 (100)	*-*	2 (100)	-	2 (100)	-
*aacA4 + aadB +* *aadA1* (1)	1 (100)	-	1 (100)	-	1 (100)	-	1 (100)	-	1 (100)	*-*	1 (100)	-	1 (100)	-
*aacA4 + aadB + aphA6* (1)	1 (100)	-	1 (100)	-	1 (100)	-	-	1 (100)	1 (100)	*-*	-	1 (100)	1 (100)	-
*aadB + armA + aphA6* (3)	3 (100)	-	3 (100)	-	2 (66.6)	1 (33.3)	2 (66.6)	1 (33.3)	3 (100)	*-*	3 (100)	-	2 (66.6)	1 (33.3)
*aacA4 + aacC2 + aphA6* (1)	1 (100)	-	1 (100)	-	1 (100)	-	1 (100)	-	1 (100)	*-*	1 (100)	-	1 (100)	-
*aacA4 + armA* *+ aphA6* (2)	2 (100)	-	2 (100)	-	2 (100)	-	2 (100)	-	2 (100)	*-*	2 (100)	-	2 (100)	-
*aacC2 + aacA4 + aadA1* (1)	1 (100)	-	1 (100)	-	1 (100)	-	1 (100)	-	1 (100)	*-*	1 (100)	-	1 (100)	-
*aacA4 + aadA1 + aphA6* (1)	1 (100)	-	1 (100)	-	1 (100)	-	1 (100)	-	1 (100)	-	1 (100)	-	1 (100)	-
*aacC2 + aacA4* *+ aadB + aphA6* (1)	1 (100)	-	1 (100)	-	1 (100)	-	1 (100)	-	1 (100)	-	1 (100)	-	1 (100)	-
*aacA4 + aadB +* *aadA1 + aphA6* (4)	4 (100)	-	4 (100)	-	4 (100)	-	4 (100)	-	4 (100)	*-*	4 (100)	-	4 (100)	-
*aacC2 + aacA4 + aadA1 + aphA6 + aadB* (14)	14 (100)	-	13 (92.8)	1 (7.1)	12 (85.7)	2 (14.2)	12 (85.7)	2 (14.2)	14 (100)	-	14 (100)	-	13 (92.8)	1 (7.1)
*aacC2 + aacA4 + aadA1 + aadB + armA + aphA6* (2)	2 (100)	-	2 (100)	-	2 (100)	-	2 (100)	-	2 (100)	-	2 (100)	-	2 (100)	-

**NS:** non-susceptible (resistant and intermediate resistant); **S:** susceptible.

## DISCUSSION

Overuse and misuse of antibiotics in the treatment of infections caused by *A. baumannii* has led to the emergence of MDR isolates in hospitals and health centers^24^. The spread of AME-encoding genes among the clinical isolates of *A. baumannii* is an important concern in the prescription of these traditional and effective antibiotics, as 94% of our isolates were resistant to kanamycin, gentamicin, streptomycin, and tobramycin. However, we found that netilmicin was the most effective aminoglycoside, as this antibiotic is not commonly used in the treatment of bacterial infections. This finding was similar to that of another Iranian study^1^. However, their isolates revealed an MIC_50_ ≤8 µg/mL, while in the present study it ranged from 128 µg/mL, indicating an increased resistance rate in our region. However, the MIC ranges of other clinically important aminoglycosides such as amikacin, gentamicin, and tobramycin in the present study were significantly higher than those reported in previous studies in Iran and other countries^1^ ^,^ ^5^ ^,^ ^25^, while these ranges were almost similar to those reported by Yoo Jin Cho et al. in 2009^22^. 

The molecular analysis of AME-encoding genes in the present study showed a high frequency of *aphA6, aadB, aadA1, aacA4, aacC2*, and *armA* genes, consistent with the previous studies from Iran^1^ ^,^ ^5^. Given that AME-encoding genes can spread by transferable genetic elements^18^, this high proportion would be justified. Possibly, these resistance genes can spread between different gram-negative bacteria such as *Pseudomonas aeruginosa* and Enterobacteriaceae. Further confirmation of this hypothesis can be found in another study conducted in Iran on the clinical isolates of *P. aeruginosa,* according to which *aadB* and *aacA4* were the most prevalent AME genes^26^. In a study performed by Lee et al. in Korea, the highest frequency was reported for *aphA6* (71%), *aacC1* (56%), and *aadB* (48%)^27^.

In addition, a high proportion of *aphA6* and *aadB* was reported by Akers et al. in USA in agreement with the present study; almost 42% of their isolates were collected from the burn ward and ICU. However, the resistance rates toward gentamicin and amikacin in their isolates were 96.6% and 57.1%, respectively^25^. However, *aphA6* confers resistance to amikacin and kanamycin^17^. Interestingly, 74% and 88.3% of our isolates containing *aphA6* exhibited MIC values of ≥256 µg/mL for amikacin and kanamycin, respectively. Moreover, a study carried out in Poland revealed that *aphA6* was the second most prevalent AME gene (78.7%) among 61 *A. baumannii* isolates^28^. However, *aadB,* the second most prevalent AME gene in the current study, confers resistance to gentamicin, tobramycin, and kanamycin in gram-negative bacteria^26^, while 95.8%, 93.1%, and 87.6% of our isolates containing *aadB* showed an MIC range of ≥256 µg/mL for these antibiotics, respectively. 

In addition, we found that 33% of our isolates contained *the aacA4* gene. Other research performed in the USA detected only one isolate carrying this gene from blood and wound infections that were resistant to gentamicin, tobramycin, and amikacin^25^. However, 96.9% of our *aadB*-positive isolates showed a ≥256 µg/mL MIC range for gentamicin and kanamycin; 87.8% of the isolates exhibited this MIC range for tobramycin, while in a previous study in Iran, this percentage was 26.4%^5^. It is noteworthy that this gene was reported as the second most prevalent AME gene carryied by class 1 integrons among clinical isolates of *P. aeruginosa* in Iran^26^. However, 83.6% of the aminoglycoside-resistant *A. baumannii* isolates in South Korea contained the *aacA4* gene, while their MIC ranges were 64 to greater than 1024 µg/mL^22^. Sheikhalizadeh et al. reported that 27.6% isolated exhibited the *aadA1* gene proportion, which was almost concordant with the results of the present study^5^, while another Iranian study detected 26.4%, 31%, and 54.5% exhibiting this gene among the sequence group (SG) of *A. baumannii*, 1, 2, and 3, respectively. Any isolates belonging to SG4-9 contained this resistance gene^1^. In addition, we detected that 19% of our isolates carried *aacC2*, while Akers et al. reported a 3.7% proportion of this gene^25^, and another Iranian study detected a proportion of 8.04% for this gene owing to which all isolates were non-susceptible to kanamycin^5^. However, 89.4% of our isolates containing this gene showed an MIC range of ≥256 µg/mL for spectinomycin, streptomycin, kanamycin, and tobramycin, while 100% of them exhibited this MIC range for gentamicin. Moreover, according to research by Hasani et al., this gene was detected in SG1-4^1^, while Nowak et al. did not detect this gene among their isolates^28^.

Additionally, the *armA* gene, which is an effective factor in the development of resistance to aminoglycosides in *A. baumannii*, can be placed on plasmids and frequently recognized in carbapenem-resistant isolates^18^. This gene encodes a 16S rRNA methylase, resulting in limited access of aminoglycosides to the ribosome of the bacteria and causing high-level aminoglycoside resistance (HLAR) against gentamicin, tobramycin, amikacin, and kanamycin^1^. Surprisingly, among 22 *armA*-positive *A. baumannii* isolates in this study, 21 (95.4%), 16 (72.7%), 18 (81.8%), and 21 (95.4%) isolates showed high-level resistance (MIC≥256 µg/mL) to gentamicin, tobramycin, amikacin, and kanamycin, respectively, with an MIC_50_≥128 μg/mL. Considering that most isolates in the present study were MDR, and a high proportion of strains harboring the AME genes was detected, the simultaneous presence of carbapenem-resistance genes and AME genes in *A. baumannii* has been proven^18^ ^,^ ^28^; this assumption may also be true for our isolates. However, 75-97% of our isolates were resistant to carbapenems and other β-lactams. Other studies from South Korea, Iran, and North America have reported armA production by *A. baumannii* ^1^ ^,^ ^27^ ^,^ ^29^. Additionally, other researchers have revealed the role of the *armA* gene in high-level resistance to amikacin and gentamicin^22^ ^,^ ^30^.

In addition to the material presented, the most important problem observed in our study was the simultaneous presence of aminoglycoside resistance genes. We detected 22 gene profiles, while Nowak et al. detected only 3 combinations of AME genes from 61 carbapenem-resistant and aminoglycoside non-susceptible *A. baumannii* isolates^28^. Our most prevalent combinations were *APH(3')-VIa*+*ANT(2")-Ia* (39 isolates) with 95-100% resistance rates against aminoglycosides and *AAC(3)-IIa*+*AAC(6')-Ib*+*ANT(3")-Ia*+*APH(3')-VIa*+*ANT(2")-Ia* (14 isolates) of which 93-100% were resistant to aminoglycosides. The common point between our study and the study by Nowak et al. was the presence of *aphA**6*** among most of the isolates. However, Akers et al. detected 16 AME gene profiles, of which 12 (75%) isolates had a combination of these genes^25^. The most prevalent (38/107 isolates) combination of their study included *APH(3')**-Ia*** +*ANT(2'')-Ia*, and 35 (92.1%) were concurrently resistant to gentamicin, tobramycin, and amikacin. Nevertheless, 85% of our *A. baumannii* isolates carried more than one AME gene, of which 52 (61.1%) contained 2 AME genes concurrently and most of them were resistant to all tested aminoglycosides. Moreover, we found that as the number of AME genes increased, the likelihood of resistance to aminoglycosides, especially gentamicin, tobramycin, streptomycin, and kanamycin, increased. Due to the higher proportion of strains harboring the AME genes, especially *aph,* it may be better to use phosphotransferases and acetyltransferase inhibitors such as the bovine antimicrobial peptide indolicidin, as previously reported^31^, in combination with aminoglycosides in our region, Iran. 

## CONCLUSIONS

High-level aminoglycoside MIC ranges in isolates with the simultaneous presence of AME and ArmA-encoding genes indicated the importance of these genes in resistance to aminoglycosides in *A. baumannii.* However, it seems that the selection of the appropriate antibiotic based on antimicrobial susceptibility testing and the use of combination therapy would be effective in overcoming this problem in such countries. Therefore, it is necessary to collect data from monitoring studies for the prevention, treatment, and control of the infections caused by this microorganism.
